# General Characteristics and Design Taxonomy of Chatbots for COVID-19: Systematic Review

**DOI:** 10.2196/43112

**Published:** 2024-01-05

**Authors:** Wendell Adrian Lim, Razel Custodio, Monica Sunga, Abegail Jayne Amoranto, Raymond Francis Sarmiento

**Affiliations:** 1 National Telehealth Center National Institutes of Health University of the Philippines Manila Manila Philippines

**Keywords:** COVID-19, health chatbot, conversational agent in health care, artificial intelligence, systematic review, mobile phone

## Abstract

**Background:**

A conversational agent powered by artificial intelligence, commonly known as a chatbot, is one of the most recent innovations used to provide information and services during the COVID-19 pandemic. However, the multitude of conversational agents explicitly designed during the COVID-19 pandemic calls for characterization and analysis using rigorous technological frameworks and extensive systematic reviews.

**Objective:**

This study aims to describe the general characteristics of COVID-19 chatbots and examine their system designs using a modified adapted design taxonomy framework.

**Methods:**

We conducted a systematic review of the general characteristics and design taxonomy of COVID-19 chatbots, with 56 studies included in the final analysis. This review followed the PRISMA (Preferred Reporting Items for Systematic Reviews and Meta-Analyses) guidelines to select papers published between March 2020 and April 2022 from various databases and search engines.

**Results:**

Results showed that most studies on COVID-19 chatbot design and development worldwide are implemented in Asia and Europe. Most chatbots are also accessible on websites, internet messaging apps, and Android devices. The COVID-19 chatbots are further classified according to their temporal profiles, appearance, intelligence, interaction, and context for system design trends. From the temporal profile perspective, almost half of the COVID-19 chatbots interact with users for several weeks for >1 time and can remember information from previous user interactions. From the appearance perspective, most COVID-19 chatbots assume the expert role, are task oriented, and have no visual or avatar representation. From the intelligence perspective, almost half of the COVID-19 chatbots are artificially intelligent and can respond to textual inputs and a set of rules. In addition, more than half of these chatbots operate on a structured flow and do not portray any socioemotional behavior. Most chatbots can also process external data and broadcast resources. Regarding their interaction with users, most COVID-19 chatbots are adaptive, can communicate through text, can react to user input, are not gamified, and do not require additional human support. From the context perspective, all COVID-19 chatbots are goal oriented, although most fall under the health care application domain and are designed to provide information to the user.

**Conclusions:**

The conceptualization, development, implementation, and use of COVID-19 chatbots emerged to mitigate the effects of a global pandemic in societies worldwide. This study summarized the current system design trends of COVID-19 chatbots based on 5 design perspectives, which may help developers conveniently choose a future-proof chatbot archetype that will meet the needs of the public in the face of growing demand for a better pandemic response.

## Introduction

### Background

The COVID-19 pandemic is regarded as one of the most critical public health crises to have affected societies worldwide [1,2]. In December 2019, the first human cases of COVID-19 were reported in Wuhan, China [3]. A few months later, in March 2020, the World Health Organization (WHO) declared the COVID-19 outbreak a global pandemic [4]. Since then, it has caused massive disruptions and catastrophes not only in the health care sector but also in education, transportation, the economy, and politics [1,5]. During this period, we observed a paradigm shift toward digital transformation and a surge in the use of web-based technologies to ensure safe access and provision of needed services [6,7]. The worldwide effects of the COVID-19 pandemic, especially in the health sector, have caused health authorities such as the WHO to turn to digital technologies, such as the use of chatbots [8]. Early in the pandemic, chatbots were considered an instant resource about the latest COVID-19 health advice to the public [9]. Chatbots are conversational agents powered by artificial intelligence (AI) that enable interaction between end users and machines, with the intervention of natural language processing (NLP) [6,7,10,11]. Currently, chatbots are already in use to tackle various health concerns, particularly in the realm of mental health, which has the most extensive literature available [8]. Chatbots can also currently perform many labor-intensive tasks at lower costs across other fields, such as customer service, pedagogy, and personal assistance [12]. During a pandemic, however, chatbots can be particularly useful for informing people, promoting behavior change, offering mental health assistance, and diagnosing and tracking symptoms [8]. Therefore, a surge in the use of COVID-19–related chatbots occurred around the world. Many of these COVID-19 chatbots were implemented for public health responses and information sharing, such as the WHO Health Alert via WhatsApp and Facebook [12] and the HealthBuddy of the WHO Regional Office for Europe [13]. Other uses of chatbots at the time of the COVID-19 pandemic were in the field of education, such as Iggy and Pounce [14]; business and employment, such as Benefits Bot [15]; mental health, such as Woebot [16]; and customer service, such as the Rapid Response Virtual Agent [17].

The existing literature investigated the design characteristics of chatbots in general, including their frequency of interaction and consequential design implications [18-20]. Notably, the design of a chatbot could affect user engagement, which in turn could affect its usefulness to its intended audience [18]. It is also critical to note that the design of chatbots evolves with time to perform new functions [19]. Despite this, we noticed a lack of comprehensive characterization and analysis of the chatbots explicitly designed to address the negative effects of the COVID-19 pandemic. Prior chatbot reviews mainly covered conversational agents developed before the pandemic [4,11,21]. Given the recent surge in COVID-19–related chatbot development and deployment, we believe that an up-to-date review that evaluates the characteristics and design of chatbots deployed in response to the pandemic is crucial. Analyzing the design of these technologies and their innovative applications can track recent developments in chatbot technology and assist chatbot developers and implementers in making design decisions for their conversational agents.

### Objectives

To address this gap, we aimed to determine the general characteristics of the existing COVID-19 chatbots worldwide. We also intended to examine the system blueprints of these conversational agents using the adapted and modified design taxonomy framework by Nißen et al [21].

This chatbot design framework was based on a mixed methods research study, in which 120 pre–COVID-19 chatbots with various purposes were analyzed to develop a design taxonomy. The framework characterized relationships among users and chatbots with different time horizons based on 5 design perspectives (eg, *temporal profile, appearance, intelligence, interaction,* and *context),* 22 design dimensions, and 61 design characteristics [21] (Table 1). In the interest of our work, we did not cover the *front-end user interface*, which is 1 of the 22 design dimensions in the design taxonomy by Nißen et al [21]. The *front*-*end user interface* dimension addresses user access, which can already be covered in the description of the chatbot’s general characteristics. Using this chatbot design framework, we will be able to see the design trend of COVID-19 chatbots.

**Table 1:** Modified design taxonomy for COVID-19 chatbots.

Layer, design perspective, and design dimensions				Design characteristics
**Chatbot**				
	**Temporal profile**			
		Time horizon	Short term, medium term, long term, and life long	
		Frequency of interactions	1-time only and multiple times	
		Duration of interaction	Short, medium, and long	
		Consecutiveness of interactions	Unrelated and related	
	**Appearance**			
		Role	Expert, facilitator, and peer	
		Communication style	Task oriented, and socially or chat oriented	
		Avatar representation	Disembodied and embodied	
	**Intelligence**			
		Intelligence framework	Rule-based, hybrid, and artificially intelligent	
		IQ	Rule-based knowledge only, text understanding, and other alternative sources	
		Personality adaptability	Principal self and adaptive self	
		Socioemotional behavior	Not present and present	
		Service integration	None, external data, media resources, and multiple	
**Chatbot and user**				
	**Interaction**			
		Communication modality	Text only and text+voice	
		Interaction modality	Graphical and interactive	
		User assistance design	Reactive, proactive, and reciprocal	
		Personalization	Static and adaptive	
		Additional human support	None and yes	
		Gamification	Not gamified and gamified	
**User**				
	**Context**			
		Application domain	Business, health care, education, and daily life	
		Motivation or purpose	Productivity, entertainment, utility, informational, and coaching	
		Collaboration goal	Nongoal oriented and goal oriented	

## Methods

### Search Strategies

We compiled published papers and journals on COVID-19 chatbots, targeting the social facets of the pandemic response, such as health, education, and lifestyle management, as described in our objectives. We examined databases such as PubMed, Scopus, CINAHL, ProQuest, as well as Google Scholar and ResearchGate to help us capture the gray literature. We also used the following search string for this systematic review: (“Covid” OR “Covid-19” OR “Covid19” OR “coronavirus” OR “2019nCoV” OR “SARS-COV-2”) AND (“Chatbot” OR “conversational agent” OR “virtual assistant”).

### Study Selection

**Figure 1:**
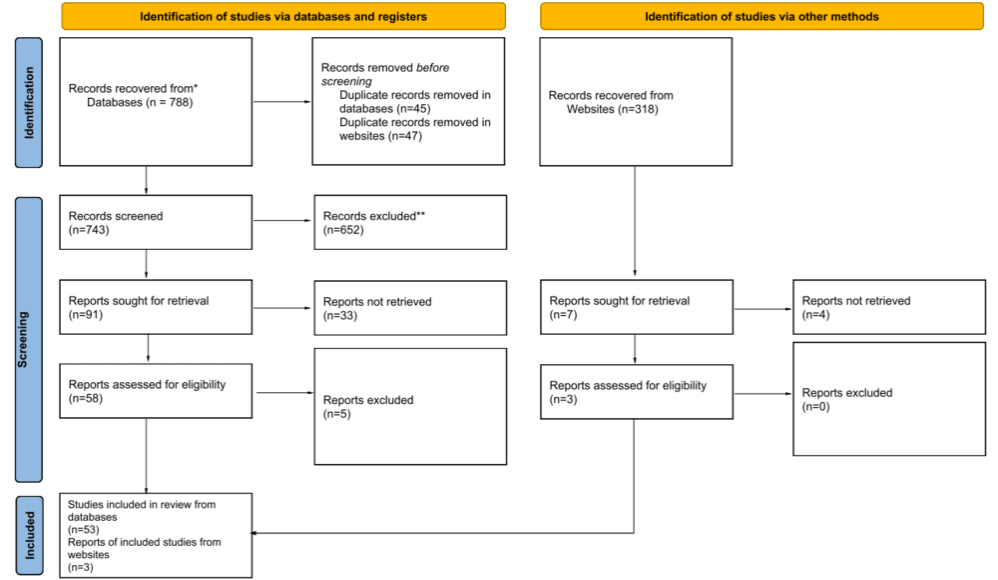
Process flowchart of study selection. PRISMA (Preferred Reporting Items for Systematic Reviews and Meta-Analyses) 2020 flow diagram for new systematic reviews which included searches of databases, registers, and other sources.

We used the PRISMA (Preferred Reporting Items for Systematic Reviews and Meta-Analyses) guidelines for this systematic review (Multimedia Appendix 1). The results of our study selection are shown in Figure 1. After eliminating duplicates and screening titles and abstracts, we developed a set of inclusion and exclusion criteria to determine the most relevant articles. We included studies that matched the following statements: (1) papers that extensively discussed the characteristics, design, and behavior of a COVID-19–specific chatbot, not limited to health; (2) papers that described a chatbot that underwent a clinical trial during the time of the pandemic; and (3) papers that detailed the development and conceptualization of a COVID-19 chatbot. Alternatively, we excluded publications that consisted of the following: (1) studies that were not written in English; (2) studies without full texts; (3) studies that were published before March 2020 and after April 30, 2022; (4) qualitative studies related to COVID-19 chatbots or conversational agents; (5) studies that mentioned chatbots related to other public health emergencies of international concern such as swine influenza, Ebola, polio, Zika virus, and Kivu Ebola; (6) other systematic reviews or meta-analyses about COVID-19 chatbots; and (7) opinion papers, commentary, news, and editorials on COVID-19 chatbots. The papers were concurrently scrutinized through intensive reading and analysis by 2 research associates and 1 senior researcher from April to June 2022. Papers published after April 30, 2022, were not included to not disrupt the paper analysis and statistics retrieved by the researchers.

## Results

### Overview

We identified 1106 papers from the databases (n=788, 71.25%) and websites (n=318, 28.75%). We then removed 92 duplicates (n=45, 49% in databases and n=47, 51% in websites) before the study screening and performed title and abstract screening. From the remaining 1014 papers in the databases and websites, we sought 98 (9.66%) for full-text retrieval. Then, of the 98 papers, we reviewed the full text of 58 (59%) from database sources and 3 (3%) from websites using the study inclusion criteria. After the final screening, we included 56 papers in this study.

### General Characteristics

The general characteristics of a COVID-19 chatbot describe its structural and technical information. These comprise the app’s name, country and continent of origin, maturity level, and the platform where it was deployed. Details of the general characteristics of COVID-19 chatbots are provided in Multimedia Appendix 2 [10,22-76]. A summary is presented in Table 2.

**Table 2:** Summary of the general characteristics of the COVID-19 chatbots (N=56).

Characteristic and category		Values, n (%)
**Region or continent**		
	Asia	24 (43)
	Europe	15 (27)
	North America	7 (13)
	Africa	4 (7)
	South America	3 (5)
	Oceania	3 (5)
**Maturity level**		
	Released	26 (46)
	Beta stage	11 (20)
	Conceptualization	10 (18)
	Alpha stage	5 (9)
	Prealpha stage	2 (4)
	Prototype	2 (4)
**Platform**		
	SMS text messaging apps	17 (30)
	Websites	16 (29)
	Android operating system	12 (21)
	Others (eg, iOS and Google cloud)	9 (16)

### Design Taxonomy

#### Temporal Profile Perspective

Table 3 shows the distribution of studies in relation to their temporal profiles, which refer to the time-dependent characteristics of a chatbot and can be further classified according to the time horizon, frequency of interaction, duration of the exchange, and consecutiveness of interactions [21]. Our results indicated that a substantial proportion of COVID-19 chatbot interactions (26/56, 46%) is categorized under the medium term, as the interaction of these chatbots can go beyond a single conversation and may last for days and weeks. With regard to frequency of interactions, more than half of the COVID-19 chatbots (31/56, 55%) interacted with their users multiple times instead of a single occurrence [21]. Regarding interaction duration, most COVID-19 chatbots had medium-length conversations with the user (22/56, 39%).

Regarding the consecutiveness of interactions, nearly half of the chatbots (25/56, 45%) could remember information from previous user interactions, whereas the remaining chatbots (8/56, 14%) were found to perform the opposite function toward past interactions with the user.

**Table 3:** Temporal profile perspective of the COVID-19 chatbots (N=56).

Design dimension and design characteristic		Values, n (%)	Study
**Time horizon**			
	Short term	11 (20)	[28,34,40,41,45,47-51,59]
	Medium term	26 (46)	[31,33,36,39,42-44,46,54,56-58,60,63,64,66-76]
	Long term	2 (4)	[35,38]
	Not stated	17 (30)	[10,22-27,29,30,32,37,52,53,55,61,62,65]
**Frequency of interaction**			
	1 time only	8 (14)	[28,34,40,41,45,47,48,59]
	Multiple times	31 (55)	[26,31,33,35,36,38,39,42,44,46,49,54,56,57,60-76]
	Not stated	17 (30)	[10,22-25,27,29,30,32,37,43,50-53,55,58]
**Duration of interaction**			
	Short	14 (25)	[28,34,40-42,45-51,57,59]
	Medium	22 (39)	[31,33,35,36,39,44,54,56,60,63,64,66-76]
	Not stated	20 (36)	[10,22-27,29,30,32,37,38,43,52,53,55,58,61,62,65]
**Consecutiveness of interactions**			
	Related	25 (45)	[26,31,33,35,36,39,48,49,54,60-68,70-76]
	Unrelated	8 (14)	[28,34,40,41,43,45,46,69]
	Not stated	23 (41)	[10,22-25,27,29,30,32,37,38,42,44,47,50-53,55-59]

#### Appearance Perspective

In Table 4, we characterize the appearance of COVID-19 chatbots (ie, the chatbot’s overall look and feel) into 3 design dimensions: *role*, *primary communication style*, and *avatar representation* [21]. Regarding chatbot roles, our results showed that most conversational agents for COVID-19 were considered *experts* (33/56, 59%). Expert chatbots have specific skills or expertise in a specific field and provide answers or reactions to the user’s query, keywords, or actions [21]. In contrast, facilitator chatbots assist users in reaching a certain objective or executing a particular task [21]. In our analysis, 27% (15/56) were facilitator COVID-19 chatbots. Finally, peer chatbots aim to be a “sparring partner” for the user, and they value socioemotional behavior as part of their design. Of the 56 chatbots, only 8 (14%) COVID-19 chatbots served as peers.

Regarding the communication style of COVID-19 chatbots, we found that 63% (35/56) of them were task oriented. Task-oriented chatbots prioritize task efficiency and achievement of goals at the most minimal cost and time possible [21]. In contrast, socially oriented chatbots prioritize social engagement and personalization of interactions with their users [21]. These comprised only 34% (19/56) of COVID-19 chatbots.

Chatbots can also have a visual or character representation (embodied avatars), or they may not have one at all (disembodied avatars) [21]. We found that more than half of the COVID-19 chatbots (32/56, 57%) did not have any visual or character representation. Only 34% (19/56) of the COVID-19 chatbots had embodied avatars.

**Table 4:** Appearance perspective of the COVID-19 chatbots (N=56).

Design dimension and design characteristic			Values, n (%)		Study
**Role**					
	Expert	33 (59)		[10,22-24,26,29-34,38,39,41,43,47,50,51,55-58,63,64,66-73,75,76]	
	Facilitator	15 (27)		[25,27,35-37,40,45,53,54,59-62,65,72]	
	Peer	8 (14)		[28,42,44,46,48,49,52,74]	
**Primary communication style**					
	Task oriented	35 (63)		[23-26,29,30,32-34,37,39,40,43,45,46,54-69,71,72,75,76]	
	Socially or chat oriented	19 (34)		[10,22,27,28,35,36,38,41,42,44,47-52,70,73,74]	
	Not specified	2 (4)		[31,53]	
**Avatar representation**					
	Disembodied	32 (57)		[10,23-26,29,30,32-34,36,37,39-41,52,56,60,61,63-75]	
	Embodied	19 (34)		[22,27,28,35,38,42,44-51,53,54,57,62,76]	
	Not specified	5 (9)		[31,43,55,58,59]	

#### Intelligence Perspective

The intelligence perspective is characterized by the functionalities of a chatbot’s inner working mechanisms, which build on an intelligence framework and quotient, personality adaptability, socioemotional behavior, and service integration [21]. As depicted in Table 5, we found that a large number of COVID-19 chatbots (26/56, 46%) were artificially intelligent. Artificially intelligent chatbots use their NLP capabilities to learn from each conversation with the user [21]. Meanwhile, a high proportion of COVID-19 chatbots (25/56, 45%) could respond to users according to set rules and by understanding an individual’s textual input.

With regard to personality adaptability, more than half of the COVID-19 chatbots (31/56, 55%) have a *principal* self-adaptability, meaning they operate on a structured flow. In contrast, a significant number of COVID-19 chatbots (24/56, 43%) were *adaptive* because these chatbots adjust their responses depending on the user input [21].

Moreover, 57% (32/56) of the COVID-19 chatbots did not demonstrate socioemotional behavior toward their clients. Among the existing COVID-19 chatbots, only 36% (20/56) could express empathic reactions to the users.

With regard to service integration, there was an equal distribution of the number of COVID-19 chatbots that were limited to only processing external data and those that could process both external data and broadcast resources (21/56, 38%).

**Table 5:** Intelligence perspective of the COVID-19 chatbots (N=56).

Design dimension and design characteristics		Values, n (%)	Study
**Intelligence framework**			
	Rule based	14 (25)	[25,27,30,32,36,37,40,46,60,61,65,69,72,74]
	Hybrid	14 (25)	[10,22,54-57,63,66-68,70,71,73,76]
	Artificially intelligent	26 (46)	[23,24,26,28,29,33-35,38,39,41-45,47-53,59,62,64,75]
	Not stated	2 (4)	[31,58]
**IQ**			
	Rule-based knowledge only	14 (25)	[25,27,30,32,34,36,40,41,43,46,65,68,69,72]
	Text understanding	14 (25)	[10,22-24,26,28,29,33,35,37-39,42,71]
	Both	25 (45)	[44,45,47-56,59-64,66,67,70,73-76]
	Not stated	3 (5)	[31,57,58]
**Personality adaptability**			
	Principal self	31 (55)	[22-27,29,30,32,34,37,39-41,43-46,53-61,65,68,69,72]
	Adaptive self	24 (43)	[10,28,33,35,36,38,42,47-52,62-64,66,67,70,71,73-76]
	Not stated	1 (2)	[31]
**Socioemotional behavior**			
	Not present	32 (57)	[10,23-26,29,30,32-34,37,39-41,43,45,55,59-69,71,72,75,76]
	Present	20 (36)	[22,27,28,35,36,38,42,44,46,48-52,54,57,58,70,73,74]
	Not stated	4 (7)	[31,47,53,56]
**Service integration**			
	None	2 (4)	[55,61]
	External data	21 (38)	[10,23-27,30-34,39,40,45,60,65,71,72,74-76]
	Media resource	3 (5)	[41,46,57]
	Multiple	21 (38)	[22,28,29,35-38,48,50,51,54,59,62-64,66-70,73]
	Not stated	9 (16)	[42-44,47,49,52,53,56,58]

#### Interaction Perspective

The domain encompasses the design dimensions of the users’ communication with the COVID-19 chatbot. As depicted in Table 6, of the 56 COVID-19 chatbots, 38 (68%) were text based, where the end user and the bot interacted and communicated through text or internet messaging. The interactive modality was the most predominant (39/56, 70%), where end users can enter free texts to communicate with a chatbot [21].

Regarding user assistance design, more than half of the COVID-19 chatbots (31/59, 54%) were reactive, meaning they only reacted to user input. Concerning personalization, most COVID-19 chatbots (36/56, 64%) were adaptive, meaning they could change their meaning and adjust their responses based on user needs and circumstances [21]. Moreover, most COVID-19 chatbots did not receive any additional human support (44/56, 79%) or have any gaming elements (42/56, 75%).

**Table 6:** Interaction perspective of the COVID-19 chatbots (N=56).

Design dimension and design characteristics		Values, n (%)	Study
**Communication modality**			
	Text only	38 (68)	[22,23,25,26,29,30,32-41,43,45-48,51,54,55,57,60-66,68,69,71,72,74,75]
	Text and voice	16 (29)	[10,24,27,28,42,44,49,50,52,53,56,59,67,70,73,76]
	Not specified	2 (4)	[31,58]
**Interaction modality**			
	Graphical	13 (23)	[22,25,27,32,36,40,46,57,62,65,68,69,72]
	Interactive	39 (70)	[10,23,24,26,28-30,33-35,37-39,41,42,44,45,47-54,56,59-61,63,64,66,67,70,71,73-76]
	Not specified	4 (7)	[31,43,55,58]
**User assistance design**			
	Reactive	30 (54)	[10,23-30,32-37,40,45,46,52,53,55,56,58-61,65,68,69,72]
	Proactive	1 (2)	[71]
	Reciprocal	23 (41)	[22,38,39,41-44,47-51,54,62-64,66,67,70,73-76]
	Not specified	2 (4)	[31,57]
**Personalization**			
	Static	19 (34)	[23-26,30,34,36,37,40,46,55,57,58,60,61,65,68,69,72]
	Adaptive	36 (64)	[10,22,27-29,32,33,35,38,39,41-45,47-54,56,59,62-64,66,67,70,71,73-76]
	Not specified	1 (2)	[31]
**Additional human support**			
	Yes	10 (18)	[10,39,43,53,58,62,67,68,72,73]
	No	44 (79)	[22-30,32-38,40-42,44-52,54-57,60,61,63-66,69-71,74-76]
	Not specified	2 (4)	[31,59]
**Gamification**			
	Gamified	6 (11)	[42,47,48,54,57,73]
	Not gamified	42 (75)	[10,22-30,32-41,43-46,53,55,56,60,62-72,74-76]
	Not specified	8 (14)	[31,49-52,58,59,61]

#### Context Perspective

Context refers to the motivation of users to interact with the chatbot, and it is reflected in the application domain, the user motivation to engage with the chatbot, and the collaboration goal of the chatbot. As depicted in Table 7, the vast majority of COVID-19 chatbots were in the health care domain (46/56, 82%). Consequently, we found 20% (11/56) of the COVID-19 chatbots in the education domain, and there was a smaller percentage of chatbots designed activities of daily living (6/56, 11%). Among all the COVID-19 chatbots, 11% (6/56) fell under multiple application domains.

We also found that most COVID-19 chatbots (35/56, 63%) were purposely designed to provide information to the user. Our results also showed that there was an almost equal distribution of COVID-19 chatbots designed for utility (18/56, 32%) and coaching purposes (15/56, 27%). However, some COVID-19 chatbot researchers designed their conversational agents for productivity (6/56, 11%) and entertainment (4/56, 7%) purposes. It is also noteworthy that several COVID-19 chatbots (16/56, 29%) were designed for multiple purposes.

**Table 7:** Context perspective of the COVID-19 chatbots (N=56).

Design dimension and design characteristic			Values, n (%)		Study
**Application domain^a^**					
	Health care	46 (82)		[10,22-29,31-46,48,50,51,55,58,59,61,63-76]	
	Education	11 (20)		[27,30,44,47,48,53,56,57,60,62,65]	
	Daily life	6 (11)		[42,44,46,49,52,54]	
**Motivation or purpose^b^**					
	Productivity	6 (11)		[48,54,60,62,65,75]	
	Entertainment	4 (7)		[44,49,54,57]	
	Utility	18 (32)		[10,22,25,31,33,34,37-40,48,54,59,61,62,67,68,76]	
	Informational	35 (63)		[1-3,6,7,10,12-19,21-68,73,75-77]	
	Coaching	15 (27)		[23,27,35,36,42,44,46,52,54,57,61,70,72-74]	
**Collaboration goal**					
	Goal oriented	56 (100)		[10,22-76]	

^a^In total, 11% (6/56) of the chatbots fall under multiple application domains.

^b^In total, 29% (16/56) of the chatbots are designed to for multiple purposes.

Finally, all the COVID-19 chatbots were goal oriented, which means that the chatbots helped users achieve a predefined goal [78].

## Discussion

### Overview

Chatbots have a huge impact on augmenting the public health response worldwide because of their wide accessibility, functionality, practicality, and fast dissemination of information in addressing the debilitating effects of COVID-19 [79]. This systematic review successfully classified 56 COVID-19 chatbots worldwide based on their general characteristics and systems design, which was based on the design taxonomy by Nißen et al [21]. After further analysis of the collated COVID-19 chatbots, our research yielded the following findings.

### General Characteristics

Regarding the general characteristics of the existing COVID-19 chatbots, we found that most studies on COVID-19 chatbot design and development were conducted in Asia and Europe. This indicates the need to conduct and implement more studies on COVID-19 chatbot research in other continents. Our research also revealed that most chatbots were accessible on websites, internet messaging apps (eg, Facebook Messenger, Viber, Telegram, WeChat, and LINE), and the Android operating system. This implies the ease of development and accessibility of web-based and internet messaging app chatbots as well as the popularity of social media in this generation [76]. Future research should focus on the design and development of pandemic-related chatbots for less popular native platforms such as iOS and Google Cloud to cater to a wider variety of people. Studies on the evaluation of the effectiveness of such chatbots in mitigating the negative effects of a pandemic should also be conducted to enrich the body of knowledge on chatbot designs.

### Design Taxonomy

#### Overview

A recent scoping review pooled the existing conversational agents combating the negative effects of COVID-19 and classified them based on their applications [6]. In this study, we updated the number of existing COVID-19 chatbots and supplemented the current literature with the design trends of existing COVID-19 conversational agents. We adopted and modified the chatbot design taxonomy of Nißen et al [21] and classified each COVID-19 chatbot based on 5 design perspectives.

#### Temporal Profile Perspective

From the temporal profile perspective, nearly half of the COVID-19 chatbots (26/56, 46%) interacted with the user for several weeks (medium term). This implies that most COVID-19 chatbots did not establish a deep, long-lasting relationship and companionship with the user, which is observed in chatbots with a long-term time horizon [21]. *COOPERA* (COVID-19: Operation for Personalized Empowerment to Render Smart Prevention and Care Seeking), for example, provides individualized self-care support, real-time follow-up of the users’ health condition, and the epidemiological situation in the users’ area for up to several weeks [31].

Moreover, COVID-19 chatbots tended to interact with their users for >1 time, which was seen in 55% (31/56) of these chatbots. For instance, Indonesian researchers deployed *ARIGAselfCareNursingBot*, a teleassessment chatbot, for 5 months in a wound care clinic [56]. Furthermore, 45% (25/56) of COVID-19 chatbots could remember information from previous user interactions. *COOPERA*, as discussed previously, could monitor users’ health conditions daily based on their previous answers about their physical and health conditions [31].

However, it is notable that a significant proportion of COVID-19 chatbot papers did not explicitly report the time horizon (17/56, 30%), frequency (17/56, 30%), duration (20/56, 36%), and consecutiveness of interactions (23/56, 41%) of their chatbot. This may show that there is a need for researchers and computer programmers to have a deeper dive into chatbot design specifications in their respective manuscripts to help future research in the identification of the most cost-efficient design for a pandemic-responsive chatbot.

#### Appearance Perspective

**Figure 2:**
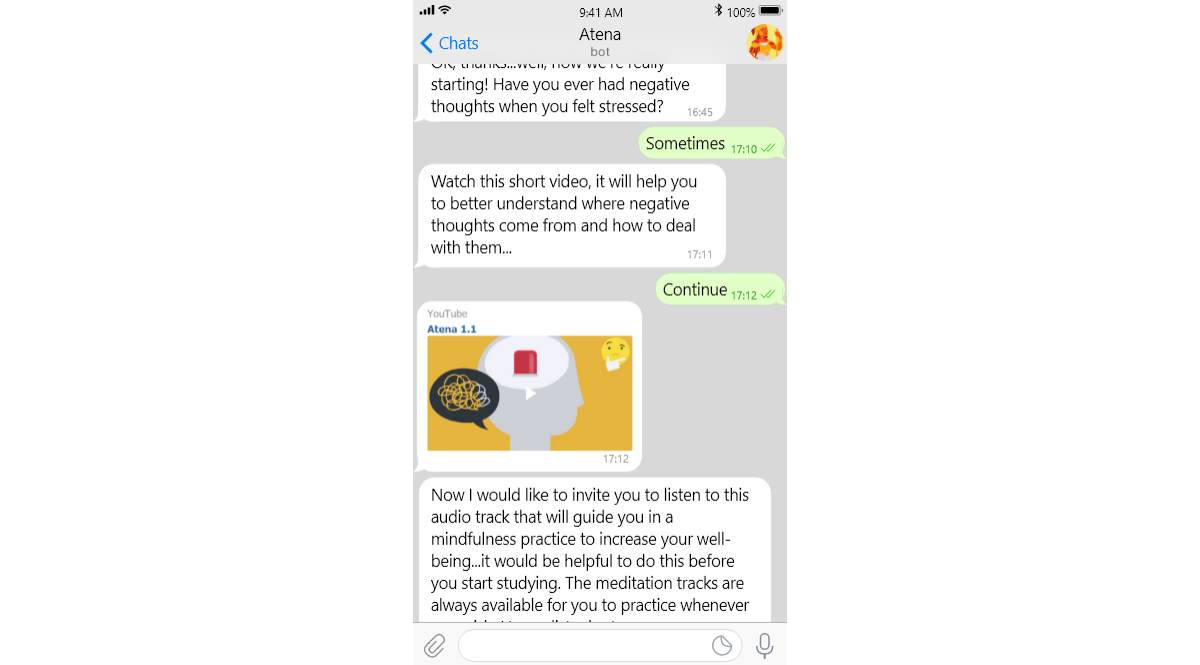
Screenshot of the Atena chatbot.

From the appearance perspective, we found that most COVID-19 chatbots (33/56, 59%) were considered experts. For example, *Chloe* provides Canadians with up-to-date information regarding COVID-19 by answering users’ inquiries about the pandemic [22]. In contrast, only 27% (15/56) of the COVID-19 chatbots assumed a facilitator role. An example of this is *Atena,* a chatbot that delivers digital mental health intervention and suggests effective coping strategies to improve the mental well-being of university students [36]. A screenshot showing a sample of conversation from the *Atena* chatbot is shown in Figure 2 [36].

Our research also found that most COVID-19 chatbots (35/56, 63%) were task oriented in terms of primary communication style. To illustrate, *Symptoma* assesses the end user’s risk of COVID-19 based on the symptoms the person inputted or answered in the textbox, without considering the user’s emotional needs [34]. This is in contrast with socially oriented chatbots, which represent only 34% (19/56) of the literature examined. An example is *Bella*, which demonstrates empathy and emotional expressions based on the user’s inputs to personalize every interaction [28].

Finally, our analysis showed that more than half of the existing COVID-19 chatbots (32/56, 57%) were disembodied, meaning they did not have any character representation. Chatbots with avatars could strengthen their social presence, which may reinforce a good social relationship with the users [79]. This finding may suggest that the immediate priority of designers at the height of the COVID-19 pandemic is to fulfill their functional efficiency while deprioritizing social engagement that may lengthen development cycles.

#### Intelligence Perspective

From the intelligence perspective, a significant number of COVID-19 chatbots (26/56, 46%) were artificially intelligent. One example is *Drever*, an educational assistant developed in Japan using an AI engine that acts as a teacher capable of answering questions from students [62]. Similarly, Real Conversation is an AI-based chatbot in India that provides COVID-19 information while assisting with customer queries [64].

Meanwhile, a high number of COVID-19 chatbots (25/56, 45%) could respond to users according to a set of rules and by understanding the textual input of the user. For example, *CoronaGo* is a health care chatbot embedded in a website that uses NLP to understand user intention but also has a rule-based system architecture to track the COVID-19 situation in India efficiently [66]. This means that most COVID-19 chatbots maximize their capabilities in understanding the users’ intentions in a timely manner, which is critical for a chatbot designed for a pandemic.

Most COVID-19 chatbots (31/56, 55%) operate on a structured flow, for example, *Chatbot-Mental Wellness*, a chatbot developed in India that provides guided activities in simple exercises, meditation, and positive affirmation [46], although nearly half (24/56, 43%) of the chatbots can adjust their responses depending on user input. An example is the *JuggleChat*, an educational assistant chatbot that addresses students’ specific needs dependent on their input [47].

Our analysis also showed that most COVID-19 chatbots (32/56, 57%) did not present socioemotional behavior to the users, whereas only 36% (20/56) of COVID-19 chatbots could express empathic reactions to the consumers. This suggests that expectations for chatbots to exhibit social behaviors that are customary in human-to-human communication should always be considered as there is a growing demand for humanlike conversational engagement styles [80]. Some empathic chatbots include *Chasey*, a character-based conversation agent for COVID-19, who responds to users with phrases such as “Don’t worry my friend, everything will be fine.” and “Get well soon, pal!” [51].

Finally, COVID-19 chatbots that are limited to only processing external data and those that can process both external data and media resources were of equal distribution (21/56, 38%). Such chatbots include *Mano*, an interactive chatbot that provides up-to-date COVID-19 information from reliable sources including, but not limited to, the WHO and predicts pandemic effects in the Mano River Union region [76], and *Aapka Chikitsak*, a medical bot that provides its users with quality information from the National Health Portal and engaging media as a means to deliver telehealth services in India during the pandemic [70].

#### Interaction Perspective

From the interaction perspective, most COVID-19 chatbots (38/56, 68%) reach out and communicate with the user through text or internet messaging and even allow the user to respond via free text inputs. This is essential as limiting interaction options may indicate a disregard for the users’ inputs [81]. One example is *Elena+*, a smartphone-based and conversational agent that delivers pandemic lifestyle care interventions that communicate with the user through textual input and is currently being expanded into Spanish and Tamil languages to reach larger audiences [35]. Another example is *Wakamola,* a chatbot that recognizes unstructured messages and is used to encourage confined individuals to modify their lifestyle practices [37].

In addition, more than half of the COVID-19 chatbots (30/56, 54%) reacted only to user input rather than steering the conversation. This may imply that chatbot designers should aim to design constructive, goal-oriented interactions [82]. Similar to a proof-of-concept study implemented at the University of Trento in Italy, *Atena* was able to react to user inputs only [36], unlike the lone proactive *Alia* that can steer conversation [71].

Moreover, most COVID-19 chatbots (36/56, 64%) had adaptive personalization. This shows that pandemic designers concentrated on the chatbots’ capacity to adapt to users’ choices, needs, and circumstances, which is consistent with an earlier study [81]. *Cory COVID-Bot*, for instance, which was developed to engage with difficult-to-reach populations directly, aims to resolve public ambiguity and uncertainty regarding community health guidelines. With its sophisticated features, an algorithm dynamically determines which questions to ask a user and when to solve them, encompassing recommendations on COVID-19 safety and behavior change interventions [38].

Furthermore, the vast majority of COVID-19 chatbots did not have any additional human support nor gamification (44/56, 79% and 42/56, 75%, respectively). These design elements for chatbot interaction show that COVID-19 chatbots are designed in such a way that most COVID-19 chatbots straightforwardly interact with the user and identify their intentions as soon as possible. Previous research suggests that conversational agents should possess a humanlike quality or assistive nature, particularly within telemedicine platforms [82]. In addition, incorporating games can help evoke personality traits and enhance thoroughness [83]. Notably, a proposed conversational agent from a university hospital in the United Kingdom targets these 2 design characteristics [73]. The in-app features will be gamified as a motivational strategy for older people in self-isolation to alleviate problems with mental health. Moreover, it is planned that this chatbot will have a social support function to facilitate cognitive behavioral therapy sessions.

#### Context Perspective

From the context perspective, almost all COVID-19 chatbots (46/56, 82%) fell under the health care application domain. We expected this because COVID-19 is a public health concern. Some of these chatbots were used for personal risk assessment [10], monitoring of COVID-19 exposure [22], and dissemination of health information regarding the pandemic [26].

Aside from health care, 20% (11/56) of COVID-19 chatbots were used in the education domain. For instance, *Student Interactive Assistant Android Application with Chatbot* is an academic chatbot developed during the COVID-19 pandemic that assists students interactively concerning their academic requirements [30]. Only a minority of COVID-19 chatbots (6/56, 11%) were used for other purposes such as assisting with daily life. In Morocco, for instance, a chatbot is being developed with the intention of aiding students daily with their mental health [52].

Our results also showed that most COVID-19 chatbots (35/56, 63%) were designed to provide information to the users. For example, a chatbot developed in France informs and educates people about COVID-19 vaccines and reduces vaccine hesitancy [32]. There were also COVID-19 chatbots designed for utility and coaching purposes, but these types comprised only 32% (18/56) and 27% (15/56) of COVID-19 chatbots, respectively. For example, a chatbot developed by the University of California was used as a COVID-19 screening tool for health employees entering their hospital before their clinical shift [40]. Regarding coaching, Managing Stress at University instructs Indian University students on stress management during the COVID-19 pandemic by providing 3 different patterns of tailored explanations (ie, belief-based, goal-based, and belief- and goal-based explanations) on students’ intentions to change the recommended behaviors [27].

Although most chatbots (40/56, 71%) served only a single purpose, there were several COVID-19 chatbots (16/56, 29%) that were multipurpose in nature. For instance, *COOPERA,* as discussed previously, can be used to monitor COVID-19 symptoms, provide self-care instructions, and suggest preventive actions to avoid infections [31].

The use of COVID-19 chatbots across numerous application domains and contexts demonstrates the pandemic’s impact on other elements of life. Nonhealth industries could investigate the use of a conversational agent to efficiently solve inquiries and issues without the need for human contact. This is critical for mitigating the rapid transmission of a global pandemic. Nevertheless, our results show that the bulk of the COVID-19 chatbots were developed to disseminate updated information regarding the COVID-19 pandemic and to track virus-related symptoms and other health-related concerns. As a result, automation of knowledge extraction from reputable sources may be required for information dissemination chatbots to be deployed efficiently and effectively as knowledge sources [78].

### Limitations

To the best of our knowledge, this is the first review to systematically analyze the general characteristics and system designs of existing COVID-19 chatbots worldwide, thereby providing developers with directions for designing chatbots in response to a pandemic. Although pioneering, this study also has its limitations. First, we limited our search to peer-reviewed studies published in English with unrestricted access to their full texts. Second, the team did not perform ancestral searches on existing scoping and systematic reviews of COVID-19 chatbots. Third, meta-analysis was not performed in this systematic review, meaning that the effectiveness of COVID-19 chatbots in mitigating the effects of the pandemic was not analyzed. We acknowledge that the latter is critical to understanding the relationship between the current design of the chatbots and their efficacy in reducing the impacts of COVID-19, which may lead to a broader perspective on the management of a global pandemic. Finally, owing to the heterogeneity of the methodology and study designs included in this review, quality assessment, risk of bias, and critical appraisal of the papers were not conducted.

### Recommendations

Our results indicate that there is a need to supplement COVID-19 chatbot research in the United States, Africa, and Australia as well as design and development studies on less well-known platforms for chatbots (eg, iOS and Google Cloud). A meta-analysis of existing COVID-19 chatbots is also recommended because there is a lack of studies evaluating the effectiveness of such chatbots. In addition, researchers and computer programmers are suggested to use the chatbot design framework by Nißen et al [21] for the purposes of having a uniform framework and for the identification of a cost-efficient design for a chatbot designed for a pandemic. Our findings also indicate that COVID-19 chatbots are used in various application areas aside from health care, which suggests the need for future investigations into other potential uses of chatbots during a pandemic. Moreover, aside from the design-centric approach toward the development of such chatbots, future studies must also evoke the assessment of user satisfaction and engagement with this type of chatbot as these are essential factors to consider for better uptake and use of these types of technology. Accordingly, it is imperative to appraise the influence of these types of conversational agents in the clinical practice guidelines and expert-client relationships [84]. In addition, our results show that several studies have “not stated” elements in multiple categories of the framework. This is because we noticed that the authors did not report the entire design specifications of their chatbot. It would be ideal for the authors to include all the intricate details of the design and development of chatbots to help future research on the most cost-efficient design for a chatbot made to address the negative effects of a pandemic.

### Conclusions

This paper describes the general characteristics and design taxonomy of the existing COVID-19 chatbots. The conceptualization, development, implementation, and use of these conversational agents emerged to mitigate the effects of a global pandemic in societies worldwide. This study successfully summarized the current system design trends of COVID-19 chatbots based on 5 design perspectives using a chatbot design taxonomy, which may serve as a starting point for helping health and nonhealth chatbot communities on how to build and deploy a future-proof chatbot archetype that would meet the needs of the public amid the growing demand for better services as part of a more robust pandemic response. This would also direct researchers to explore methods on how to develop comprehensive evaluation and analytical methods for conversational agents in health care.

## Appendix

Multimedia Appendix 1 PRISMA (Preferred Reporting Items for Systematic Reviews and Meta-Analyses) flow diagram.

Multimedia Appendix 2 General characteristics of the COVID-19 chatbots.

## Abbreviations

AI: artificial intelligence
COOPERA: COVID-19: Operation for Personalized Empowerment to Render Smart Prevention and Care Seeking
NLP: natural language processing
PRISMA: Preferred Reporting Items for Systematic Reviews and Meta-Analyses
WHO: World Health Organization
