# Supplementary material for: General Characteristics and Design Taxonomy of Chatbots for COVID-19: Systematic Review
Source: J Med Internet Res. 2024 Jan 5;26:e43112. doi: 10.2196/43112 (PMC10773556; doi:10.2196/43112)
Supplement: Multimedia Appendix 2 [file jmir_v26i1e43112_app2.docx]

| **Author** |  | **Country of Origin** | **Implementation Site** | **Name of Application** | **Maturity Level** | **Region**  **Or Continent** | **Platform** |
| --- | --- | --- | --- | --- | --- | --- | --- |
| [Battineni et al. (2020)](#Battineni) | [[10]](#Battineni) | Italy | Italy | Not specified | Pre-alpha | Europe | Not specified |
| [Siedlikowski et al. (2021)](#Siedlikowski) | [[22]](#Siedlikowski) | Canada | Canada | Chloe | Released | North America | Dialogue Platform |
| [Lee et al. (2021)](#Lee) | [[23]](#Lee) | South Korea | South Korea | Not specified | Alpha | Asia | Google Cloud |
| [Pandey et al. (2022)](#Pandey2) | [[24]](#Pandey) | India | India | Satya | Released | Asia | Android |
| [Jain et al. (2022)](#Jain) | [[25]](#Jain) | India | India | Not specified | Conceptualization | Asia | Not specified |
| [Mukhtar et al. (2022)](#Mukhtar) | [[26]](#Mukhtar) | Saudi Arabia | Saudi Arabia | Not specified | Beta | Asia | Not specified |
| [Nelekar et al. (2022)](#Nelekar) | [[27]](#Nelekar) | India | An Indian University (Not specified) | mAnaging stRess at University (ARU) | Released | Asia | Website |
| [Loveys et al. (2021)](#Loveys) | [[28]](#Loveys) | New Zealand | New Zealand | Bella | Released | Oceania | Website |
| [Hong et al. (2021)](#Hong) | [[29]](#Hong) | South Korea | South Korea | Child vaccination chatbot | Released | Asia | Messaging App |
| [Sweidan et al. (2021)](#Sweidan) | [[30]](#Sweidan) | Jordan | University of Jordan | Student Interactive Assistant Android Application with Chatbot (SIAAA-C) | Released | Asia | Android |
| [Yoneoka et al. (2020)](#Yoneoka) | [[31]](#Yoneoka) | Japan | Kanagawa, Aichi, Shiga prefectures, Japan | COOPERA: COvid-19: Operation for Personalized Empowerment to Render smart prevention And care seeking | Released | Asia | Messaging App |
| [Altay et al. (2020)](#Altay) | [[32]](#Altay) | France | France | Not specified | Alpha | Europe | Not specified |
| [Hassan et al. (2021)](#Hassan) | [[33]](#Hassan) | Saudi Arabia | Saudi Arabia | Not specified | Released | Asia | Website |
| [Martin et al. (2020)](#Martin) | [[34]](#Martin) | Not specified | Germany, United Kingdom, Greece | Symptoma | Released | Europe | Website |
| [Rexhepi et al. (2022)](#Rexhepi) | [[35]](#Rexhepi) | Not specified | Switzerland,  Ireland,  UK,  United States of America (USA),  Spain,  Mexico,  Colombia | Elena+ | Released | Europe | iOS, Android |
| [Gabrielli et al. (2021)](#Gabrielli) | [[36]](#Gabrielli) | Italy | University of Trento, Italy | Atena | Released | Europe | Messaging App |
| [Asensio-Cuesta et al. (2021)](#Asensio) | [[37]](#Asensio) | Spain | A Spanish University (Not specified) | Wakamola Chatbot | Released | Europe | Messaging App |
| [Van Baal et al. (2022)](#Mantas) | [[38]](#Mantas) | Australia | Australia | Cory COVID-Bot | Released | Oceania | Messaging App |
| [Morales et al. (2021)](#Morales) | [[39]](#Morales) | Brazil | Curitiba, São Bernardo do Campo, and Catanduva , Brazil | Laura Digital Emergency Room | Released | South America | Website, Messaging App |
| [Judson et al. (2020)](#Judson) | [[40]](#Judson) | USA | University of California, San Francisco Health, USA | Not specified | Released | North America | Website |
| [Morse et al. (2020)](#Morse) | [[41]](#Morse) | USA | Sutter Health, USA | Not specified | Released | North America | Website / Web-based patient portal |
| [Zhu et al. (2022)](#Zhu) | [[42]](#Zhu) | China | Wuhan, Chongqing, and Hangzhou, China | Xiaolv | Released | Asia | Messaging App |
| [Lai et al. (2020)](#Lai) | [[43]](#Lai) | USA | Mass General Brigham (MGB) Hospital, USA | MGB chatbot | Beta | North America | Website |
| [Mateos-Sanchez et al. (2022](#Mateos)) | [[44]](#Mateos) | Spain | Insolamis Association, Spain | CapacitaBOT | Beta | Europe | Android |
| [Driss et al. (2022](#Driss)) | [[45]](#Driss) | Saudi Arabia | Not specified | COVIBOT | Alpha | Asia | Not specified |
| [Raji & Maheswari,](#Harshini) 2022 | [[46]](#Harshini) | India | Chennai, Bengaluru, Mumbai, Hyderabad, Coimbatore, and Pune, India | Chatbot-Mental Wellness | Released | Asia | Messaging App |
| [Briel, 2021](#Briel) | [[47]](#Briel) | USA | Prolific Academic, USA | JuggleChat | Beta | North America | Website |
| [El Hefny et al. (2021)](#ElHefny) | [[48]](#ElHefny) | Germany | German University in Cairo, Egypt | Academic Chatbot and COVID-19 Chatbot | Beta | Europe | Messaging App |
| [Fu et al. (2021)](#Fu) | [[49]](#Fu) | Japan | Japan | ERICA | Beta | Asia | Android; Android Robot |
| [Khattab, 2021](#Khattab) | [[50]](#Khattab) | Egypt | Egypt | Ask Nameesa | Released | Africa | Messaging App |
| [El Hefny et al. (2021)](#ElHefny1) | [[51]](#ElHefny) | Germany | Not specified | Chasey | Beta | Europe | Messaging App |
| [Salhi et al. (2021)](#Salhi) | [[52]](#Salhi) | Morocco | Not specified | Not specified | Alpha | Africa | Not specified |
| [Al-madi et al. (2021)](#Almadi) | [[53]](#Almadi) | Jordan | Al-Zaytoonah Private University of Jordan, Jordan | Intelligent Arabic Chatbot System (Z.A.Y.C.H.A.T.) | Prototype | Asia | Website, Web Application |
| [Valtonina and Hu, 2021](#Valtolina) | [[54]](#Valtolina) | Italy | Not specified | Charlie | Beta | Europe | Android |
| [Amer et al. (2021)](#Amer) | [[55]](#Amer) | Egypt | Not specified | Not specified | Conceptualization | Africa | Not specified |
| [Ariga et al. (2021)](#Ariga) | [[56]](#Ariga) | Indonesia | African Social Research Initiative Wound Care, North Sumatra | ARIGAselfCareNursingBot | Beta | Asia | Android, Messaging App |
| [Holt-Quick et al. (2021)](#Holtquick) | [[57]](#Holtquick) | New Zealand | Two local secondary schools, New Zealand (Not specified) | Headstrong | Released | Oceania | Messaging App |
| [Alkmim et al. (2021)](#Alkmim) | [[58]](#Alkmim) | Brazil | State of Minas Gerais: Divinópolis and Teófilo Otoni, Brazil | Not specified | Released | South America | Website, Messaging App |
| [Kim, 2021](#Kim) | [[59]](#Kim) | USA | Not specified | Not specified | Alpha | North America | Android |
| [Du et al. (2021)](#Du) | [[60]](#Du) | Hong Kong | A public university (Not specified), Hongkong | Learning Buddy | Released | Asia | Learning Management System |
| [Aminuddin et al. (2021)](#Aminuddin) | [[61]](#Aminuddin) | Malaysia | Universiti Teknologi MARA,  Malaysia | Athlete Trainer Bot (ATBOT) | Conceptualization | Asia | iOS, Android |
| [Ito et al. (2021)](#Ito) | [[62]](#Ito) | Japan | Kanazawa Institute of Technology, Japan | Drever | Pre-alpha | Asia | Learning Management System |
| [Chalaguine and Hunter, 2021](#Chalaguine) | [[63]](#Chalaguine) | United Kingdom | Online (Not specified) | None | Beta | Europe | Website |
| [Patgar et al. (2021)](#Patgar) | [[64]](#Patgar) | India | Online (Not specified) | Re-Co (Real Conversation) | Released | Asia | Messaging App |
| [Oruche et al. (2021)](#Oruche) | [[65]](#Oruche) | USA | Not specified | Viruda Chatbot Assistant | Conceptualization | North America | Website |
| [Pandey et al. (2021)](#Pandey) | [[66]](#Pandey) | India | India | Coronago | Released | Asia | Android, Website |
| [Hossain et al. (2020)](#Hossain) | [[67]](#Hossain) | Bangladesh | Bangladesh | iSecure | Beta | Asia | Android |
| [Erazo et al. (2020)](#Erazo) | [[68]](#Erazo) | Ecuador | Medical Department at Universidad de las Américas, Ecuador | Telebot (COVID Assistant) | Released | South America | Website |
| [Maniou and Veglis, 2020](#Maniou) | [[69]](#Maniou) | Cyprus and Greece | Cyprus and Greece | COVINFO Reporter Chatbot | Released | Europe | Website |
| [Bharti et al. (2020)](#Bharti) | [[70]](#Bharti) | India | Rural and urban areas (Not specified), India | Aapka Chikitsak | Conceptualization | Asia | Google Cloud Platform |
| [Bahja et al. (2020)](#Bahja) | [[71]](#Bahja) | United Kingdom | United Kingdom | Alia | Prototype | Europe | Not specified |
| [Gross et al. (2020)](#Gross) | [[72]](#Gross) | Switzerland | University Hospital in Zurich, Switzerland | CAir Chatbot | Conceptualization | Europe | iOS, Android |
| [Meinert et al. (2020)](#Meinert) | [[73]](#Meinert) | United Kingdom, France, Sweden | United Kingdom, France, Sweden | Not specified | Conceptualization | Europe | Mobile digital health app but did not specify as to what platform |
| [Rodrigo et al. (2021)](#Rodrigo) | [[74]](#Rodrigo) | Philippines | Philippines | None | Conceptualization | Asia | Messaging App |
| [Singh et al. (2022)](#Singh) | [[75]](#Singh) | India | India | None | Conceptualization | Asia | Not specified |
| [Fagbolu et al. (2021)](#Fagbolu) | [[76]](#Fagbolu) | Sierra Leone, Guinea, and Liberia | Sierra Leone, Guinea, Liberia | COVID-19 Mano | Conceptualization | Africa | Messaging App |
